# Overexpression of FZD7 promotes glioma cell proliferation by upregulating TAZ

**DOI:** 10.18632/oncotarget.13292

**Published:** 2016-11-11

**Authors:** Xia Qiu, Jianguo Jiao, Yidong Li, Tian Tian

**Affiliations:** ^1^ Department of Neurology, The First Affiliated Hospital of Zhengzhou University, Zhengzhou, People's Republic of China; ^2^ Department of Nuclear Medicine, The First Affiliated Hospital of Zhengzhou University, Zhengzhou, People's Republic of China; ^3^ Institute of Clinical Medicine, The First Affiliated Hospital of Zhengzhou University, Zhengzhou, People's Republic of China; ^4^ Department of Medicine, Shangqiu Medical School, Shangqiu, Henan Province, China

**Keywords:** FZD7, TAZ, glioma, cell proliferation, prognosis

## Abstract

Gliomas are the most prevalent type of primary brain tumors in adults, accounting for more than 40% of neoplasm in the central nervous system. Frizzled-7 (FZD7) is a seven-pass trans-membrane Wnt receptor that plays a critical role in the development of various tumors. In this study, we detected high-level FZD7 expression in glioma and its overexpression was associated with advanced tumor stage. *In vitro* functional assays showed that forced overexpression of FZD7 promoted proliferation of gliomas cells, whereas knockdown of endogenous FZD7 significantly suppressed proliferation ability of these cells. In a xenograft assay, FZD7 was also found to promote the growth of glioma cells. We further found that FZD7 could activate transcriptional coactivator with PDZ-binding motif (TAZ), and TAZ was required for FZD7 to promote cell proliferation in glioma. Furthermore, the univariate analysis of survival shows that glioma patients with high FZD7 expression have a shorter survival. In conclusion, our findings demonstrate that FZD7 may promote glioma cell proliferation via upregulation of TAZ.

## INTRODUCTION

Gliomas are the most prevalent type of primary brain tumors in adults, accounting for more than 40% of neoplasm in the central nervous system [1, 2]. Gliomas are divided into several histological subtypes, including astrocytomas, ependymomas and oligodendrogliomas [3, 4]. Astrocytomas are the largest group of gliomas, representing 75% of all gliomas [2, 5]. According to the World Health Organization grading criteria, gliomas are histologically classified into 4 malignancy grades (I–IV) [6]. *Glioblastoma* (GBM, grade IV) is the most biologically aggressiv malignant gliomas with a median survival of less than 1 year from the time of diagnosis [1, 7, 8]. Despite advances in surgical resection, adjuvant radiotherapy and chemotherapy, treatment of GBM still remains one of the most challenging tasks in clinical oncology. The molecular biology of GBM is highly heterogeneous [9–12]. Until now, the molecular pathogenesis underlying the development of GBM is still not well understood. Therefore, understanding the molecular mechanism involved in tumor biology is urgently needed to identify novel therapeutic targets for GBM.

Studies have revealed that dysregulation of canonical Wnt signaling is involved in the development of various human tumors including glioma [13, 14]. Activation of the canonical Wnt pathway is transduced through the cell-surface receptor complex Frizzled (FZD) and the lipoprotein receptor-related protein (LRP) 5/6, which then initiates the β-catenin signaling cascade [15, 16]. Frizzled-7 (FZD7) is a seven-pass trans-membrane Wnt receptor and highly conserved *throughout* evolution, from *Hydra to humans* [17]. It has been recently reported that FZD7 is upregulated in several human cancers, and also appears to promote tumorigenesis and cancer progression [18–24]. However, the role of FZD7 in the development of glioma remains largely unexplored.

In this study, high FZD7 expression was detected in glioma, and its overexpression promoted glioma cell proliferation *in vitro* and *in vivo*. Furthermore, FZD7 directly regulated the expression of transcriptional coactivator with PDZ-binding motif (TAZ) (also known as WW domain containing transcription regulator 1, WWTR1) [25, 26] in glioma cells, suggesting that FZD7 may promote glioma cell proliferation via upregulation of TAZ.

## RESULTS

### FZD7 is overexpressed in glioma and its level is positively correlated with advanced tumor stage

**Figure 1:**
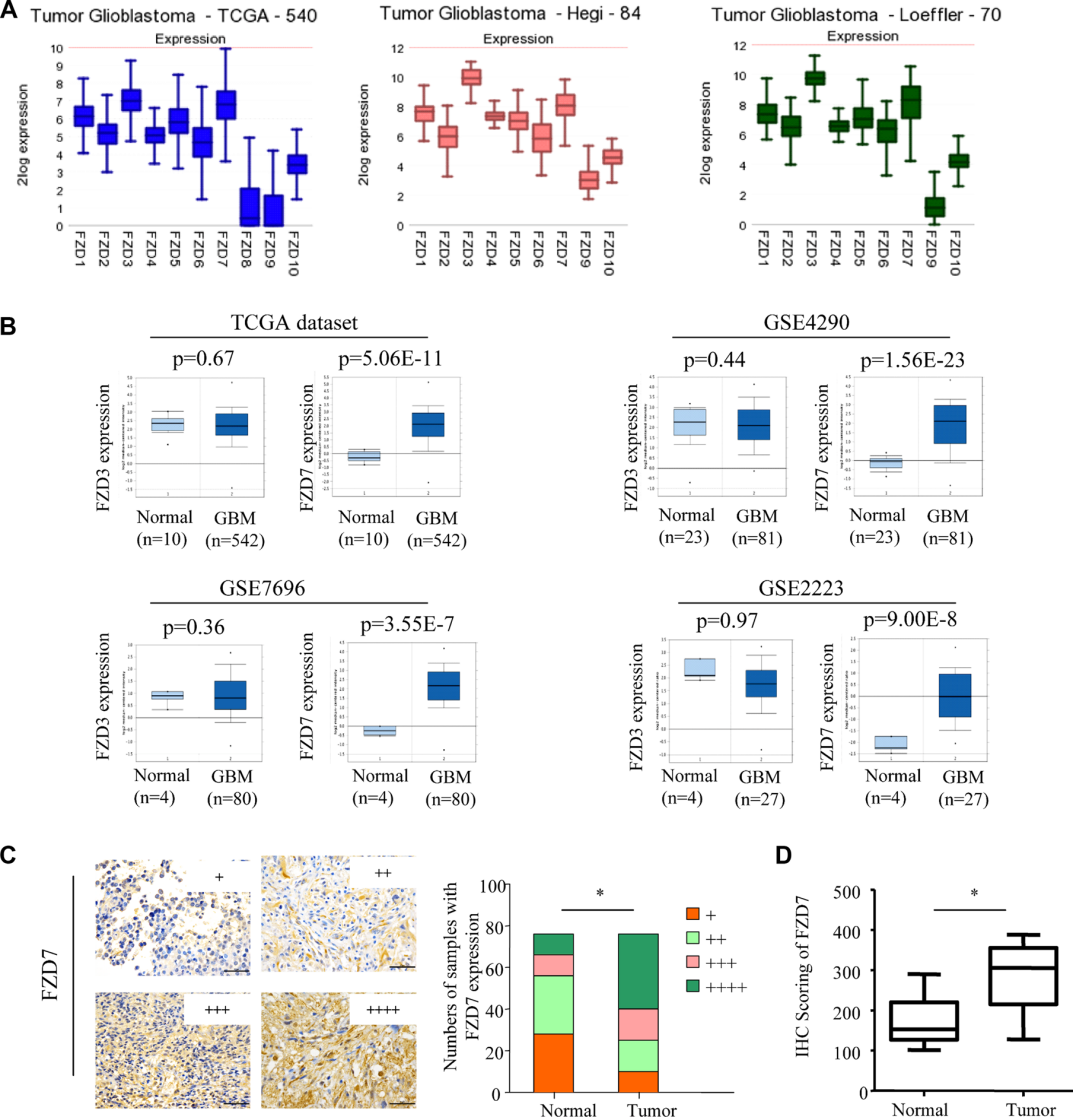
Expression of FZD7 in glioma (**A**)Ten different FZD receptors (FZD1-10) in three GBM datasets through the R2 microarray analysis and visualization platform (http://r2.amc.nl). (**B**) The expression of FZD7 is increased in glioma when compared with adjacent non-tumor tissue. All data, including *p*-values, were calculated from ONCOMINE. (**C**) Immunohistochemical study of FZD7 expression in 76 glioma tissues (Tumor) and its adjacent non-tumor tissues (Normal). Expression levels of FZD7 were scored semi-quantitatively based on the percentage of positive cells according to the following scale: +, <25%; ++, 25–49%; +++, 50–74%; and ++++, 75–100%. Scale bars = 50 μm. (**D**) Expression levels of FZD7 were scored by H-score. *p < 0.05.

To investigate the role of FZD7 in glioma development, we tested the expression of FZD7 in glioma. First, we examined mRNA expression of the ten different FZD receptors (FZD1-10) in three GBM datasets through the R2 microarray analysis and visualization platform (http://r2.amc.nl). All the three GBM datasets showed that, among the ten different FZD receptors, FZD3 and FZD7 were the most predominantly expressed FZD receptors in GBM (Figure 1A). Next, we investigated the expression of FZD3 and FZD7 by querying the ONCOMINE database [27]. In four microarray expression studies [28–31], the expression of FZD7 mRNA is significantly higher in GBM than that in the adjacent non-tumor tissues; The range ofFZD7 mRNA *increase* was 2.4- to 5.1- fold (Figure 1B). However, expression of FZD3 didn't show significant difference between GBM and the adjacent non-tumor tissues (Figure 1B). We further examined the FZD7 expression in 76 glioma tissues and its adjacent non-tumor tissues using immunohistochemisty. Results showed that expression of FZD7 was significantly higher in tumor tissue than that in the adjacent non-tumor tissues (Figure 1C).

In addition, the correlation between expression pattern of FZD7 in glioma and their clinicopathological characteristics was also studied (Table 1). Results revealed that FZD7 overexpression was significantly correlated with higher tumor stage (*p* = 0.001). About 70% (28 of 40) of high-grade gliomas (grades III & IV) were found to overexpress FZD7. No significant association between FZD7 and age, gender, tumor size, Karnofsky performance score (KPS), differentiation, treatment options was found (*p >* 0.05; Table 1). Isocitrate dehydrogenase (IDH1) mutations in amino acid position 132 were found in 35/76 cases and there was no association between FZD7 and IDH1 mutation status (*p* = 0.15; Supplementary Figure S1).

**Table 1:** Clinicopathological characteristics of patients with glioma according to the expression of FZD7

Characteristics	FZD7 expression*		*p* value
	Low (*n* =38)	High (*n* = 38)	
Age (years)			0.64
< 45	17	15	
≥ 45	21	23	
Gender			0.35
Female	18	14	
Male	20	24	
Tumor size			0.81
< 5cm	23	22	
≥ 5cm	15	16	
KPS			0.11
< 90	18	25	
≥ 90	20	13	
Stage			< 0.01
I & II	25	11	
III & IV	13	27	
Chemotherapy			0.23
Yes	22	27	
No	16	11	
Radiotherapy			0.78
Yes	29	30	
No	9	8	
Resection			0.31
Partial	7	4	
Subtotal	11	17	
Total	20	17	

Abbreviation: KPS, Karnofsky performance status.

* The median H-score of FZD7 was used as the cut-off to divide the study cohort into high expression and low expression groups.

### FZD7 promotes glioma cell proliferation *in vitro* and *in vivo*

**Figure 2:**
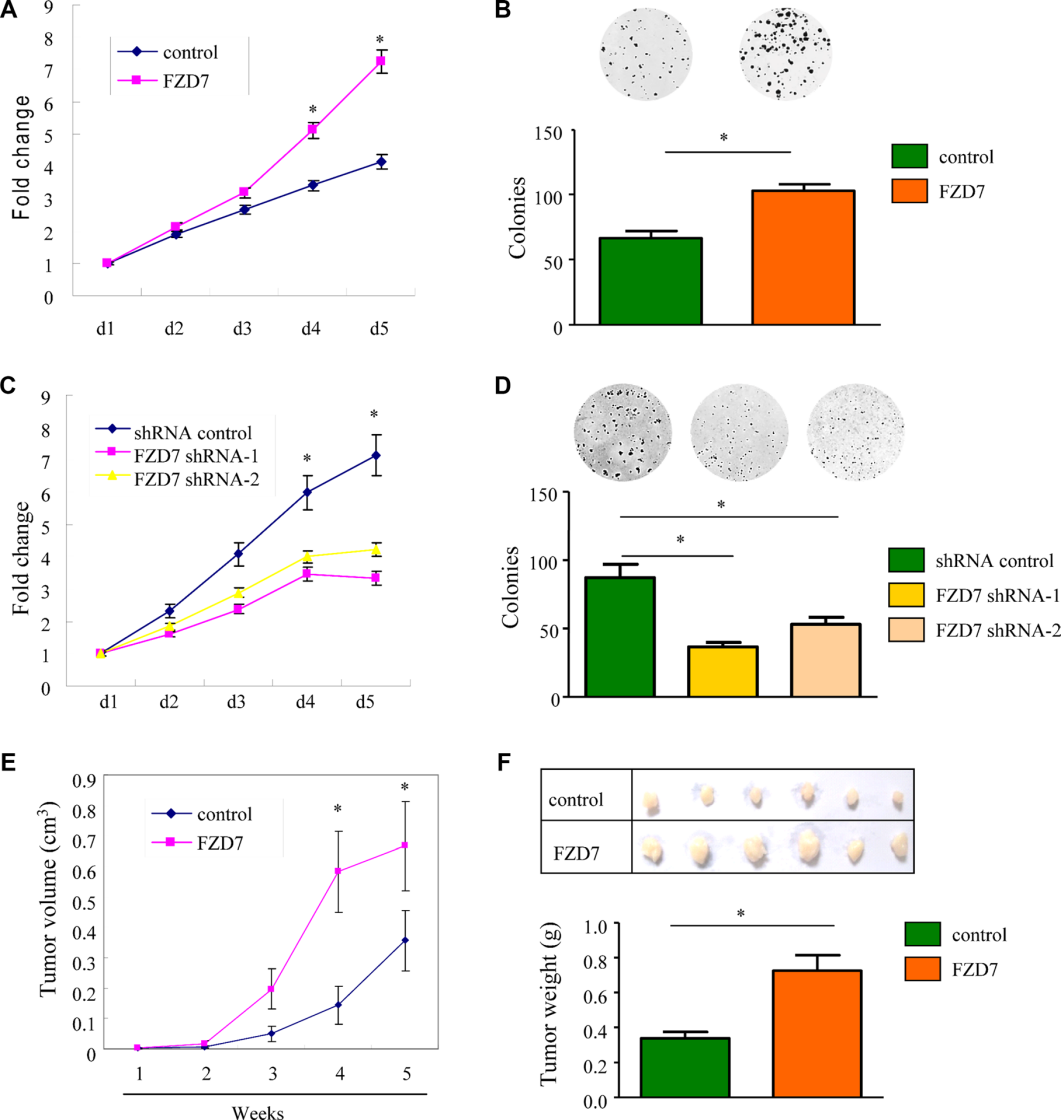
FZD7 promotes glioma cell proliferation *in vitro* and *in vivo* (**A**, **B**) MTT growth assays (A) and colony formation assay (B) of U-87MG cells stably transfected either with FZD7 or control plasmids. (**C**, **D**) MTT growth assays (C) and colony formation assay (D) of U-251MG cells stably transfected either with FZD7 shRNA or scramble control. All experiments were performed in triplicate; bars, s.e.m.; *p < 0.05. (**E**) Tumor size of subcutaneous xenografts measured every week. The tumor volume was calculated using the formula: (Length × Width^2^)/2. (**F**) Representative subcutaneous tumor xenografts generated in mice 5 weeks after inoculation (upper panel), and the weight of the tumors (lower panel). Bars, s.e.m.; *p < 0.05.

To evaluate the functional role of FZD7 in glioma, we examined the effect of FZD7 overexpression on the proliferation of U-87MG and U-251MG cells. These cells were stably transfected with either FZD7 or control vector plasmids. Cell proliferation was assessed by MTT *growth assay* and colony formation assay. According to the MTT results, the proliferation rate of glioma cells overexpressing FZD7 was significantly higher than that of vector control cells; more FZD7-transfected cells were observed at 4 and 5 days after plating (*p < 0.05, Figure 2A and Supplementary Figure S2A). Colony formation assay in U-87MG cells showed that the numbers of colonies formed within vector control and FZD7 group were 66 ± 6 and 103 ± 5, respectively (*p < 0.01, Figure 2B). We also obtained similar results in U-251MG cells (Supplementary Figure S2B). To further test whether FZD7 is required for the proliferation of glioma cells, we silenced FZD7 in glioma cells using lentivirus-mediated shRNA interference. The MTT assay demonstrated that the number of FZD7-knockdown cells was significantly lower than the number of U-251MG cells transfected with scrambled control (Figure 2C). Inhibition of endogenous FZD7 resulted in reduced colony numbers compared to the scrambled control group revealed by colony formation assay (Figure 2D). The numbers of colonies formed in shRNA control, FZD7 shRNA-1 and FZD7 shRNA-2 group were 87 ± 10, 37 ± 3 and 53 ± 5, respectively (*p < 0.01, Figure 2D). Taken together, these results indicated that FZD7 promoted glioma cell proliferation *in vitro*.

We further confirmed the above findings *in vivo* in xenograft tumor model. U-87MG cells stably overexpressing FZD7 or vector alone were injected subcutaneously into two groups of nude mice (*n* = 6). As expected, tumor growth curve showed that tumors derived from FZD7 group grew more rapidly than those from the vector control group. Five weeks after injection, the tumor volume of FZD7 group was 0.69 ± 0.14 cm^3^, whereas the tumor volume of control group was 0.36 ± 0.09 cm^3^ (*p < 0.05, Figure 2E). Moreover, mean tumor weight at the end of the experiment was significantly higher in the group overexpressing FZD7 (0.73 ± 0.17 g) compared to that in the control group (0.34 ± 0.07 g; *p < 0.05, Figure 2F). Furthermore, we tested the correlation of FZD7 expression with Ki67, a cellular marker for proliferation, in glioma patients. As shown in Supplementary Figure S3, the Ki-67 proliferation index (%) was higher in FZD high-expression group. These results demonstrated that overexpression of FZD7 promoted the proliferation of glioma cells *in vivo*.

### FZD7 upregulates TAZ in glioma cells

**Figure 3:**
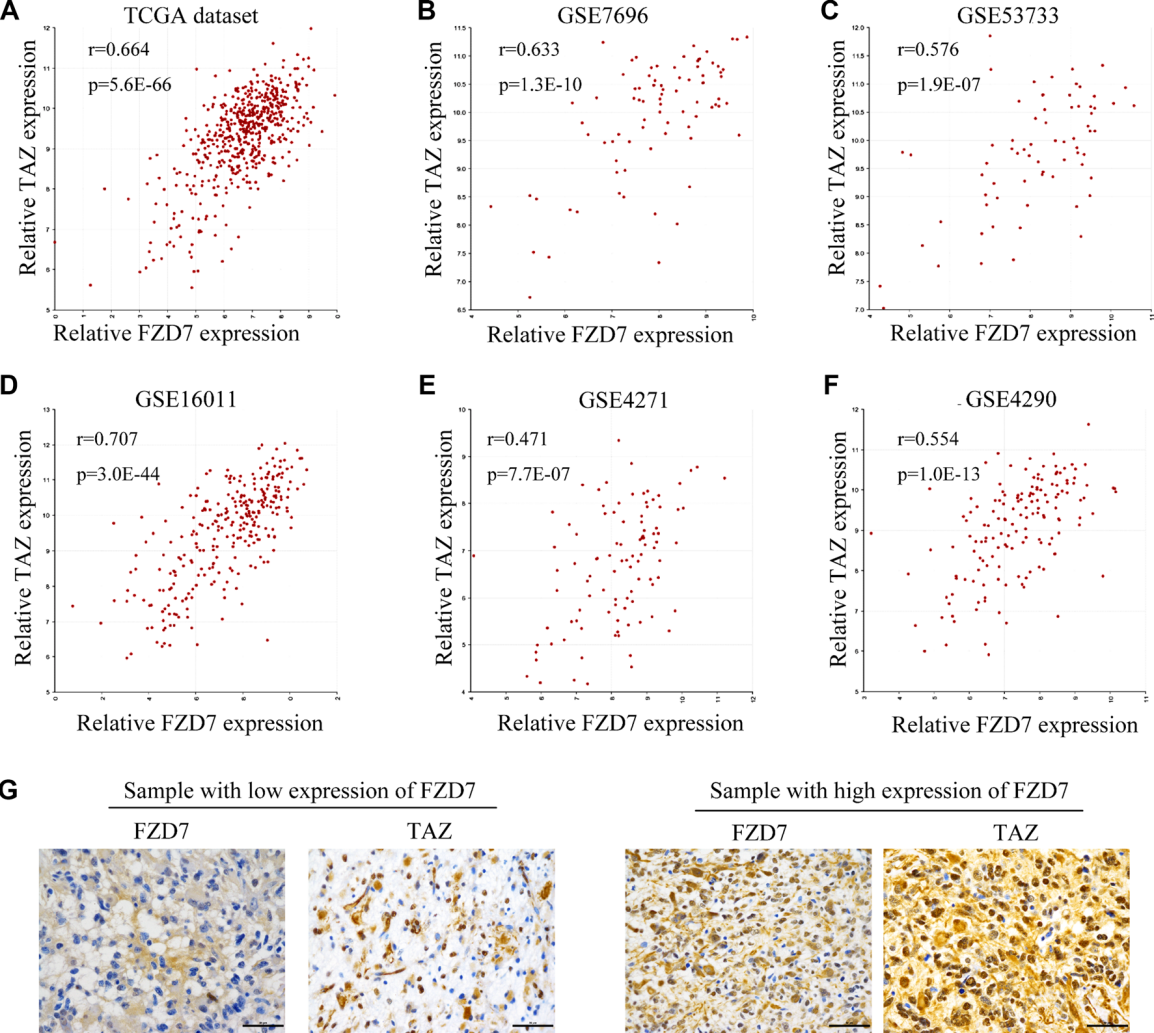
FZD7 correlates with TAZ in human glioma The correlation analysis of FZD7 and TAZ were performed in TCGA (**A**), GSE7696 (**B**), GSE53733 (**C**), GSE16011 (**D**), GSE4271 (**E**), and GSE4290 dataset (**F**) using the gene correlation module of the R2 microarray analysis and visualization platform. (**G)** The correlation between FZD7 and TAZ expression in glioma samples by immunohistochemisty analysis. Scale bars = 50 μm

To study the underlying molecular mechanisms by which FZD7 promotes proliferation of glioma cells, we analyzed the downstream targets of FZD7. A coexpression analysis of FZD7 was performed using the TCGA dataset of GBM, which showed that TAZ is one of the genes most significantly associated with FZD7 (r = 0.664, *p* = 5.6E-66; Figure 3A and Supplementary Table S1). To further confirm these findings, we examined the expression of FZD7 and TAZ in five other independent glioma microarray datasets. As a result, positive correlation between FZD7 and TAZ was confirmed in multiple datasets. (GSE7696: r: 0.633, *p* = 1.3E-10; GSE53733: r: 0.576, *p* = 1.9E-07; GSE16011: r=0.707, *p* = 3.0E- 44; GSE4271: r=0.471, *p* = 7.7E-07; and GSE4290: r = 0.554, *p* = 1.0E-13; Figure 3B–3F). Moreover, immunohistochemisty analysis of the *expression* patterns of FZD7 and TAZ in glioma samples demonstrated a significant correlation between them (Figure 3G). The representative example of FZD7 and TAZ staining in the adjacent non-tumor brain tissue was shown in Supplementary Figure S4.

**Figure 4:**
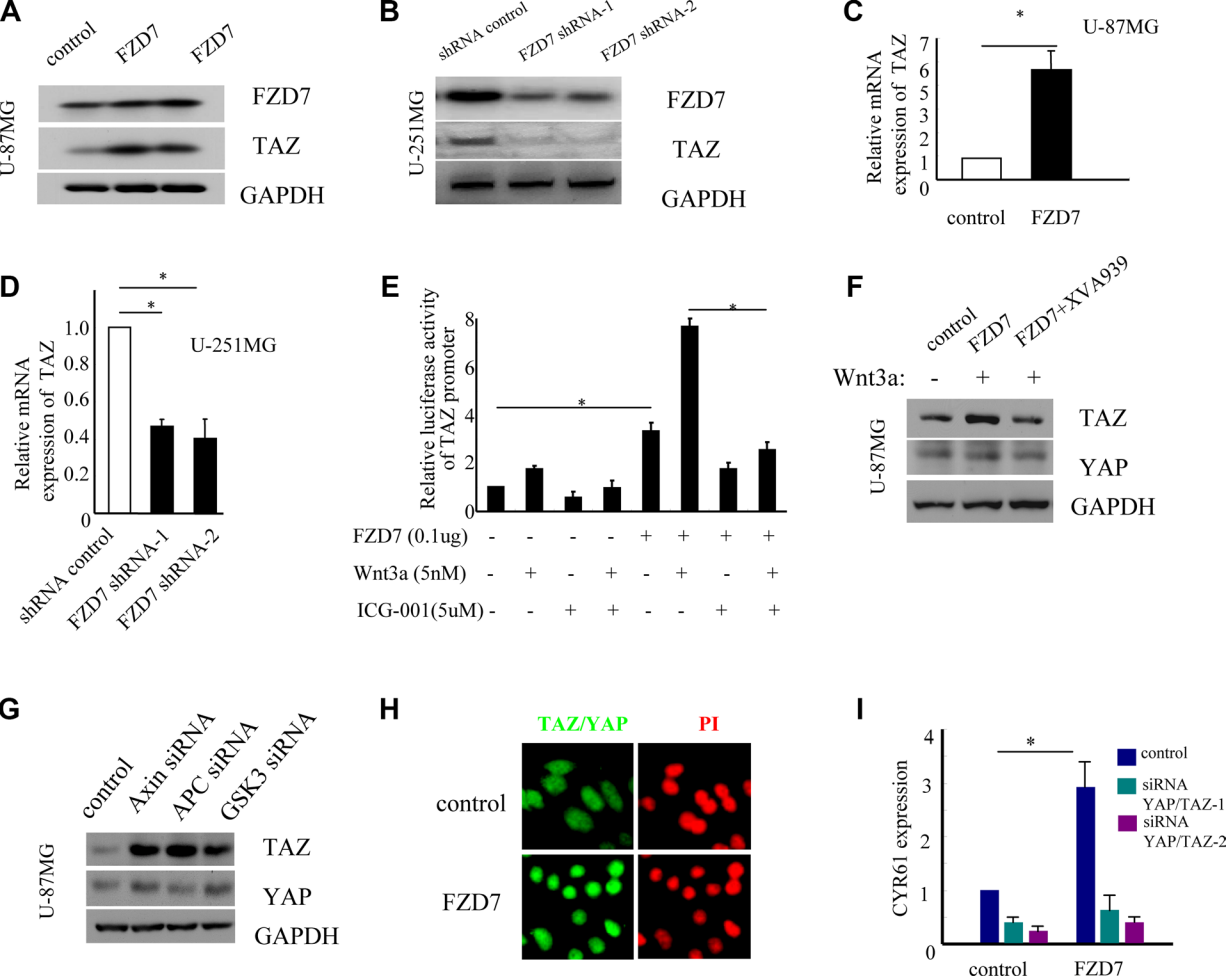
FZD7 activates YAP/TAZ in glioma cells (**A**, **C**) FZD7 upregulates the expression of TAZ protein (A) and mRNA (C) in U-87MG cells. GAPDH was used as an internal control. (**B**, **D**) Knockdown of endogenous FZD7 decreases the expression of TAZ at protein (B) and mRNA (D) level in U-251MG cells. GAPDH was used as an internal control. (**E)** FZD7 significantly enhances the luciferase activity of TAZ promoter reporter in U-87MG cells. Cells were transfected with expression vectors (empty vector or FZD7), the human TAZ promoter pGL3-TAZ and renilla reniformis luciferase (Promega, Madison, WI, USA). Cells were then cultured for 24 hours with or without ICG-001 (5μM) or Wnt3a (5nM). After that time, cells were collected and luciferase activities were measured using the Dual Luciferase Reporter Assay System (Promega, Madison, WI, USA).(**F**) U-87MG cells transduced with FZD7 or control vector were treated with Wnt3a and/or XVA939 (1uM), and then examined the protein changes of TAZ and YAP. (**G**) Knockdown of endgenous expression of Axin, APC, or GSK3, and then examined the protein changes of TAZ and YAP. (**H**) The localization of YAP/TAZ was determined by. immunofluorescence with anti-YAP/TAZ-specific antibody. (**I)** U-87MG cells transduced with FZD7 or control vector were transfected with siRNAs targeting YAP and TAZ, and then the expression of CYR61 mRNA were determined by quantitative PCR. All experiments were performed in triplicate; bars, s.e.m.; *p < 0.05.

It's well known that TAZ is a transcriptional co-activator playing a key role in regulation of organ size, tissue homeostasis and tumorigenesis [32–34]. Previous studies have demonstrated that TAZ is a downstream effector of the Wnt signaling pathway [35–38]. Therefore, we hypothesized that FZD7 might activate TAZ in glioma cells. The immunoblot assay showed that TAZ protein levels were significantly higher in U-87MG cells transduced with FZD7 than that in cells with vector control (Figure 4A), whereas in U-251MG cells with FZD7 knockdown, the expression of TAZ was dramatically reduced (Figure 4B). To determine whether FZD7 regulates the expression of TAZ at the transcription level, we analyzed TAZ expression by quantitative PCR analysis. Results showed that TAZ mRNA level increased at least 5-fold in U-87MG cells transduced with FZD7, while its level decreased significantly in U-251MG cells with FZD7 knockdown (*p < 0.05, Figure 4C and 4D). We next evaluated whether FZD7 could regulate the activity of TAZ promoter through β-catenin/TCF. U-87MG cells were transfected with TAZ promoter reporter in the presence of FZD7-expression plasmid and/or Wnt3a. Study data showed that the luciferase activity of TAZ promoter reporter was significantly enhanced by FZD7 and Wnt3a, and decreased after the disruption of β-catenin/CBP interaction by ICG-001 (*p < 0.05, Figure 4E). Previous report showed that Wnt signaling regulates TAZ in a way that depends on the β-catenin destruction complex [39]. Therefore, we sought to determine whether FZD7 regulates TAZ through this pathway in U-87MG cells. As shown in Figure 4F, Wnt3a/FZD7 increased TAZ expression; however, after reactivated the β-catenin destruction complex by a small molecule XAV939, the expression of TAZ decreased significantly. On the contrary, suppression of Axin, APC, or GSK3 induced TAZ stabilization (Figure 4G). YAP is the paralogue of TAZ, and biology of YAP and TAZ are closely associated with Hippo, Wnt, G protein-coupled receptor (GPCR), transforming growth factor beta (TGFβ) signaling etc [25, 32, 40]. However, Wnt3a or FZD7 had no effect on the protein expression of YAP. We further examined whether FZD7 affect the localization of YAP/TAZ. The results showed that forced overexpression of FZD7 led to YAP/TAZ nuclear accumulation with YAP/TAZ-dependent induction of their transcriptional target CYR61 (Figure 4H and 4I). To summarize, our data suggested that FZD7 could upregulate and activate TAZ in glioma cells.

### FZD7 promotes glioma cell proliferation via upregulation of TAZ

**Figure 5:**
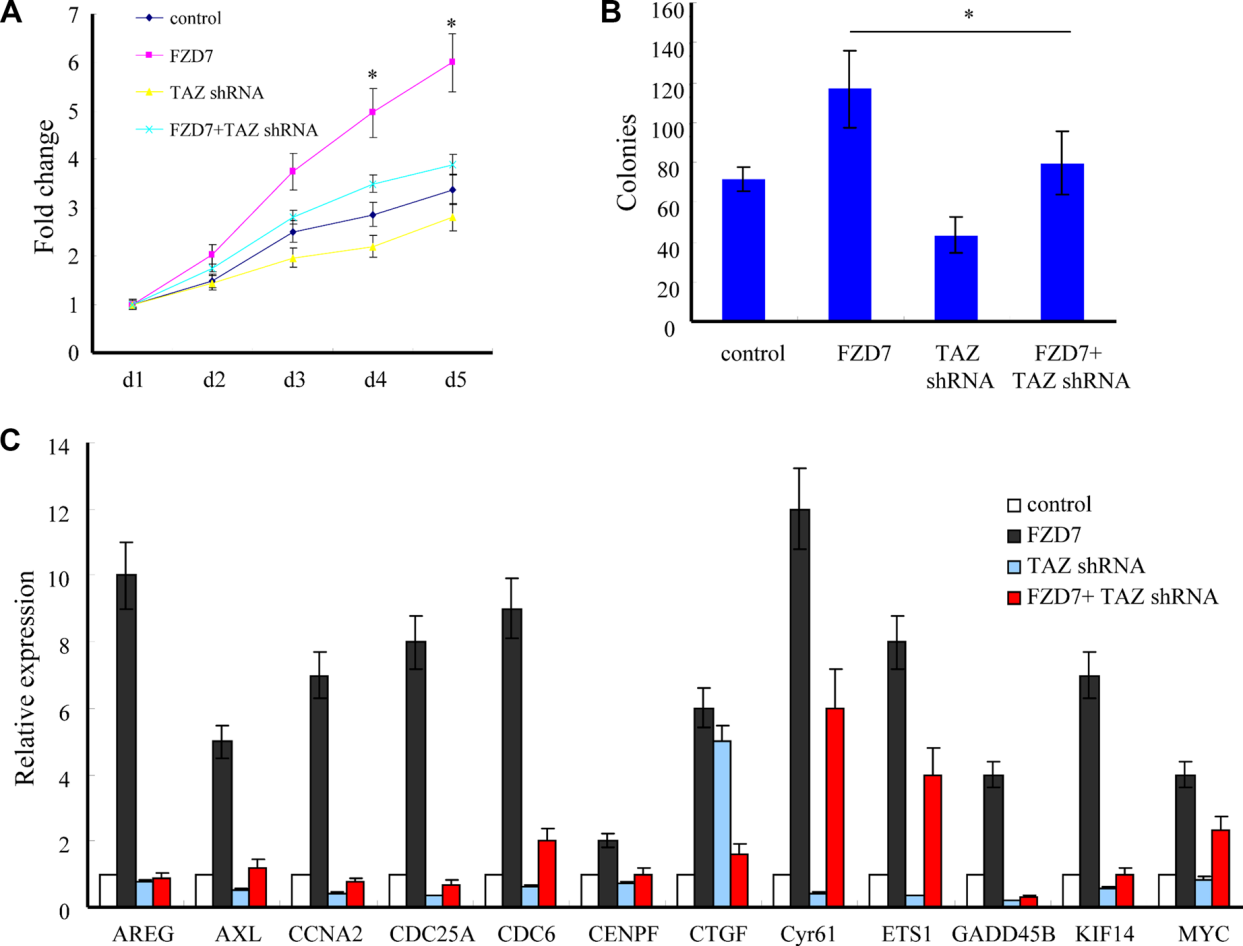
FZD7 promotes glioma cell proliferation through TAZ (**A**, **B**) Knockdown of TAZ significantly attenuated the growth advantage conferred by FZD7 in U-87MG cells as determined by both MTT (A) and colony formation assays (B). (**C**) Quantitative PCR showed that silencing of TAZ reduced the expression of FZD7 regulated genes such as *AREG, CCNA2* and *Cyr61* etc. All experiments were performed in triplicate; bars, s.e.m.; *p < 0.05

To determine whether FZD7 promotes proliferation of glioma cells through upregulation of TAZ, we knocked down the endogenous TAZ in U-87MG cells overexpressing FZD7. According to the MTT results, knockdown of TAZ significantly attenuated the growth advantage conferred by FZD7 in U-87MG cells (*p < 0.05, Figure 5A). The colony formation assay also showed that knockdown of endogenous TAZ dramatically suppressed the growth advantage conferred by FZD7 in U-87MG cells. The colony numbers of vector control group, FZD7 group and FZD7+TAZ shRNA group are (71 ± 6), (116 ± 20) and (79 ± 16), respectively (*p < 0.05, Figure 5B). We also showed that silencing of TAZ reduced the expression of FZD7 regulated genes such as *AREG, CCNA2* and *CYR61* etc. (Figure 5C). Taken together, these results suggest that TAZ is essential for FZD7-induced glioma cell proliferation.

### High expression of FZD7 associated with a poor prognosis in patients with glioma

**Figure 6:**
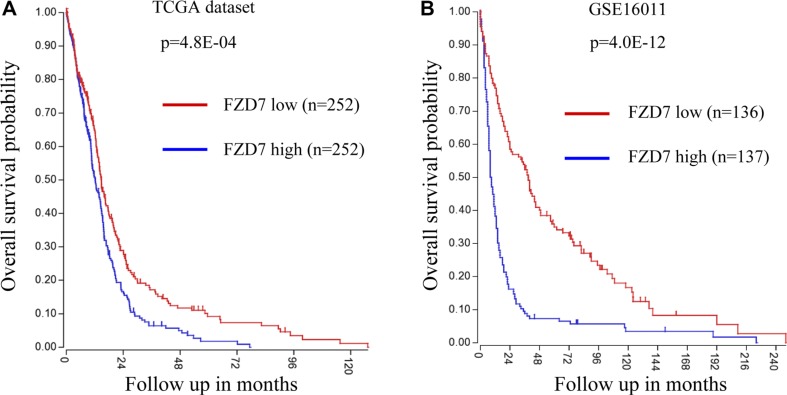
Univariate analysis of survival shows that High FZD7 expression indicates a poor prognosis for glioblastoma patients (**A**, **B**) Kaplan–Meier analysis of glioblastoma patients in TCGA (*n* = 504) and GSE16011 (*n* = 273) datasets and revealed that patients with high expression of FZD7 had a worse overall survival.

We next analyzed the clinical outcome in glioblastoma patients in two datasets: TCGA (*n* = 504) and GSE16011 (*n* = 273). The univariate analysis of survival within the two datasets was performed using the Kaplan-Meier analysis module of the R2 microarray analysis and visualization platform (http://r2.amc.nl). In TCGA dataset, patients with glioblastoma were divided into FZD7 high expression group (FZD7 high; *n* = 252) and FZD7 low expression group (FZD7 low; *n* = 252). Kaplan–Meier analysis revealed that glioblastoma patients with high expression of FZD7 had a worse overall survival probability (Figure 6A; *p* = 4.8E-04). Likewise, high level of FZD7 also conferred poor prognosis for glioblastoma patients in the GSE16011 dataset (Figure 6B; *p* = 4.0E-12). Collectively, these results suggest that glioma patients with high FZD7 expression have a poor overall survival.

## DISCUSSION

Wnt/β-catenin signaling plays a crucial role in the development of various cancers [41–48]. Recently, accumulating evidence has revealed that it is involved in oncogenic activities of cell proliferation, apoptosis inhibition and cell invasion in glioma [49–53]. Frizzled proteins, classified as a distinct family of G protein-coupled receptors, are receptors for secreted Wnt proteins in the Wnt/β-catenin signaling. To date, ten Frizzled receptors have been identified in humans. FZD7, among the ten known human Frizzled receptors, is the most commonly dysregulated Wnt receptor in various cancers including colorectal cancer, breast cancer and hepatocellular carcinoma [17, 20, 54–56]. However, the role of FZD7 in the development and progression of glioma is still not fully understood. Our study demonstrates that FZD7 is overexpressed in glioma, and its overexpression contributes to glioma tumorigenesis by upregulating TAZ. The results show, furthermore, that high FZD7 expression indicates a poor prognosis in glioma patients.

TAZ is one of the key downstream effectors of the Hippo pathway, which plays an important role in cell proliferation, organ size control, and tumorigenesis [32]. TAZ has been reported involved in tumor initiation and malignant progression of diverse forms of cancer, including brain cancer, breast cancer, sarcoma and colorectal cancer [40, 57, 58]. Overexpression of TAZ is detected in GBM, and its overexpression is significantly correlated with poor differentiation in glioma [59, 60]. Moreover, TAZ has been identified as a core player in promoting cell proliferation and driving the mesenchymal differentiation of malignant glioma [59–61]. In the present study, our data suggest that FZD7 promotes glioma cell proliferation via upregulation of TAZ, and FZD7 may enhance the expression of TAZ through β-catenin/TCF-mediated transcription in glioma cells, which is consistent with the oncogenic role of TAZ in glioma previously reported [59, 62].

Recent studies indicated that FZD7 is the most commonly upregulated Wnt receptor in the Frizzled family [20]. FZD7 plays an important role in stem cell biology and cancer development and progression. In addition, various studies have shown that targeted inhibition of FZD7 displays anti-cancer activity *in vitro* and *in vivo* [20]. In this study, we show that expression of FZD7 is significantly higher in tumor tissue than that in the adjacent non-tumor tissues, and upregulated FZD is associated with advanced tumor stage in glioma. Up to now, there have been so many ways to inhibit FZD7, such as anti-FZD7 antibody, small interfering peptides or small molecule inhibitors [63]. Therefore, targeted inhibition of FZD7 may represent a novel and promising therapeutic approach for glioma, especially the advanced stage of glioma.

To conclude, our study demonstrates that FZD7 is overexpressed in glioma, which leads to increased cell proliferation through upregulation of TAZ. In addition, univariate analysis of survival indicates that glioma patients with high FZD7 expression have a poor overall survival. Our study not only yields a better understanding of the role of FZD7 in glioma, but also paves the way for novel and powerful anticancer therapeutics.

## MATERIALS AND METHODS

### Cell lines and tissue samples

U-87MG and U-251MG glioma cells (Cell bank of Chinese Academy of Sciences, Shanghai, China) were cultured in Dulbecco's modified Eagle's medium (Hyclone, Logan, UT, USA) supplemented with 10% fetal bovine serum (Hyclone, Logan, UT, USA), 0.1 mg/ml streptomycin, and 100 units/ml penicillin (Invitrogen, California, USA) in 5% CO_2_ atmosphere at 37°C. Formalin-Fixed, Paraffin-Embedded (FFPE) glioma samples were collected at the time of diagnosis at The First Affiliated Hospital of Zhengzhou University. This study was approved by the Research Ethics Committee of Zhengzhou University. Written informed consents were obtained from all patients who provided samples.

### Establishment of stable cell lines

Lentiviral production, titration, and infection were performed as previously described [64]. Briefly, lentiviral plasmids expressing FZD7 or vector control were cotransfected with pHelper plasmids in 293T cells. Lentiviral particles were harvested from the media after 48 hours of transfection, and purified with ultracentrifugation. Cells were then infected with lentiviruses encoding FZD7 or vector control. For knockdown of endogenous expression of FZD7 or TAZ, lentiviral constructs expressing FZD7 shRNA or TAZ shRNA were used, respectively. Cells were harvested at 72 hours after infection and the knockdown efficiency was evaluated by western blot analysis.

### RNA extraction and real-time PCR

Total RNA was isolated using the RNeasy mini kit (Qiagen, Germany). cDNA was prepared using the SuperScript^®^ III First-Strand Synthesis System (Invitrogen, California, USA). Quantitative PCR was performed using SYBR Green dye on an Applied Biosystems 7300 Real-time PCR system (Applied Biosystems, Foster City, CA).

### Western blot analysis

Western blot analysis was performed as previously described [64]. Briefly, cells were lysed in cold lysis buffer, proteins (20–30 μg) were resolved on SDS-PAGE, transferred onto PVDF membranes, and probed with antibodies for FZD7 (sc-293261, Santa Cruz Biotechnology), TAZ (sc-48805, Santa Cruz Biotechnology), and GAPDH (sc-32233, Santa Cruz Biotechnology) at 4°C overnight. Detection was performed with the SuperSignal West Femto Maximum Sensitivity Substrate Trial Kit (Pierce, Rockford, IL, USA). The band images were digitally captured and quantified with a FluorChem FC2 imaging system (Alpha Innotech, San Leandro, CA, USA).

### Immunohistochemistry

The FFPE sections were immunostained using the Dako EnVision™ Flex+ System (K8012; Dako, Glostrup, Denmark). Deparaffinization and epitope unmasking were carried out in a PT-Link using an EnVision™ Flex target retrieval solution (Dako, Carpinteria, CA, USA). The sections were treated with 0.3% hydrogen peroxide (H_2_O_2_) for 5 min to block endogenous peroxidase. Sections were incubated overnight at 4°C with the following antibodies: FZD7 (sc-293261, Santa Cruz Biotechnology), TAZ (sc-48805, Santa Cruz Biotechnology) and Ki-67 (ab15580, Abcam, USA). The specimens were subsequently treated with EnVision™ Flex linker mouse or rabbit (15 min), EnVision™ Flex/HRP enzyme (30 min), and 3′3-diaminobenzidine tetrahydrochloride (10 min). The samples were counterstained with hematoxylin, dehydrated and mounted on a Richard-Allan Scientific Cyto seal XYL (Thermo Scientific, Waltham, MA, USA). The sample series included appropriate positive and negative controls. Two scoring systems were used. The protein expression was scored semi-quantitatively based on the percentage of positive cells utilizing the following scale: +, < 25%; ++, 25–49%; +++, 50–74%; and ++++, 75–100%. The protein expression was assessed using a combination of the intensity and of percentage positively stained tumor cells to generate a histological score (H-score). The H-score was calculated using the following equation: H-score = ΣPi (i + 1), where i is the intensity score (which ranged 0 ∼ 3), and Pi is the percentage of stained tumor cells at each intensity (0% ∼ 100%). This formula produces a score that ranges 100 ∼ 400, where 100 indicate that 100% of tumor cells were negative and 400 indicates that 100% of tumor cells were strongly stained. The median H-score of FZD7 was used as the cut-off to divide the study cohort into high expression and low expression groups.

### IDH1 mutation analysis

Genomic DNA from FFPE samples was extracted using the QIAamp DNA FFPE Tissue Kit (Qiagen). The quality and yield of purified DNA were assessed by fluorimetry (Qubit, Invitrogen). Primers specific to IDH1 mutations in amino acid position 132 were designed and synthesized by Sangon Biotech (Shanghai, China, Supplementary Table 3). PCR was performed using standard procedures followed by direct sequencing on an ABI 3730xl automatic sequencer (PE Applied Biosystems, Foster City, CA).

### Immunofluorescence

Cells grown on chamber slides were fixed with 4% formaldehyde in Phosphate buffered saline (PBS) for 10 min, and then were permeabilized in PBS containing 0.1% Triton X-100 for 10 min, and blocked with 2% bovine serum albumin prepared in PBS for 10 min. Cells were then incubated with anti-YAP/TAZ antibody (1 μg/ml) for 1 h. FITC –conjugated second antibodies were used at a dilution of 1:100 for 45 min. Cells were washed with PBS and nuclei stained with PI (10 μg/ml) for 15 min. Images were captured using a Leica confocal laser scanning microscope (Leica Laser Technik GmbH, Heidelberg, Germany).

### MTT assay

Cells plated in 96-well plates were incubated for different periods of time and then added 20 μL of MTT (tetrazolium bromide, 5 mg/mL, GE Healthcare) into each well. After incubation for 4 h, 150 μL of DMSO was added to solubilize the crystals for 20 min at room temperature and the absorbance at 570 nm was read on an ELISA plate reader (Model 680, Bio-Rad, CA).

### Colony formation assay

Cells (2.0 × 10^3^) were seeded into 6-well plates in triplicate in 2 ml of complete growth medium. The medium was changed every three days. Two weeks later, cells were stained by 0.1% crystal violet (Sigma-Aldrich, St. Louis, MO, USA) in methanol for 10 min. Colonies (more than 50 μm diameter) were counted directly on the plate.

### Reporter assays

Gene reporter assays were performed as previously reported [65]. Cells were transfected with expression vectors (empty vector or FZD7), the human TAZ promoter pGL3-TAZ and renilla reniformis luciferase (Promega, Madison, WI, USA). Cells were then cultured for 24 hours with or without 5 μM ICG-001 or 5 nM Wnt3a. After that time, cells were collected and luciferase activities were measured using the Dual Luciferase Reporter Assay System (Promega, Madison, WI, USA), according to the manufacturer's instructions.

### Mouse xenograft model

Mouse xenograft model were performed as previously reported [65]. Briefly, the BALB/c (6–8 weeks old) athymic nude mice were purchased from Experimental Animal Center of Henan province (Zhengzhou, China). The mice were randomly distributed into two groups and subcutaneously injected in the flank regions with 1.0 × 10^6^ cells in 0.1 mL of PBS. The tumor size was measured every week with calipers. The tumor volume was calculated with the formula: (Length × Width^2^)/2. Five weeks following implantation, a tumor began to appear at the site of implantation with 0.5 to 1.0 cm^3^ in volume. Mice were euthanized by asphyxiation in a CO_2_ chamber and tumors excised using standard forceps, scissors, and surgical blades. All procedures were conducted in accordance to Animal Care and Use Committee guidelines of Zhengzhou University.

### Analysis of microarray data

Oncomine Cancer Microarray database (http://www.oncomine.org) was used to study gene expression of FZD7 in glioblastoma samples as we previously described. Gene expression data were also obtained from NCBI Gene Expression Omnibus (GEO) database (accession numbers: GSE4290 GSE7696 and GSE2223) and The Cancer Genome Atlas (TCGA) dataset. Expression data for FZD7 were log transformed, median centered per array, and the standard deviation was normalized to one per array. The correlation analysis of FZD7 and TAZ were performed in GSE7696, GSE53733, GSE16011, GSE4271, GSE4290 and TCGA dataset. The univariate analysis of survival analysis within the glioblastoma data set of the TCGA (*n* = 504) and GSE16011 (*n* = 273) was performed using the Kaplan-Meier analysis module of the R2 microarray analysis and visualization platform (http://r2.amc.nl). The cutoff value of FZD7 in TCGA and GSE16011 datasets were 114.6 (203705_s_at) and 203.8 (203706_s_at), respectively.

### Statistical analysis

All data were expressed as mean ± standard error of the mean (SEM). Between groups and among groups comparisons were conducted with Student *t* test and ANOVA, respectively. Mann-Whitney *U* test is used for nonparametric variables. The association of FZD7 expression and clinicopathological characteristics was analyzed by Chi-square or Fisher's two-tailed exact test. Statistical analysis was performed using GraphPad Prism software version 4.0 (PRISM4) (GraphPad Software Inc, LaJolla, CA), and *p* < 0.05 was considered significant.

## SUPPLEMENTARY FIGURES AND TABLE




