# Supplementary material for: Overexpression of FZD7 promotes glioma cell proliferation by upregulating TAZ
Source: Oncotarget. 2016 Nov 11;7(52):85987–99. doi: 10.18632/oncotarget.13292 (PMC5349891; doi:10.18632/oncotarget.13292)
Supplement: Supplementary file 1 [file oncotarget-07-85987-s001.pdf]

Overexpression of FZD7 promotes glioma cell proliferation by upregulating TAZ

Supplementary Materials

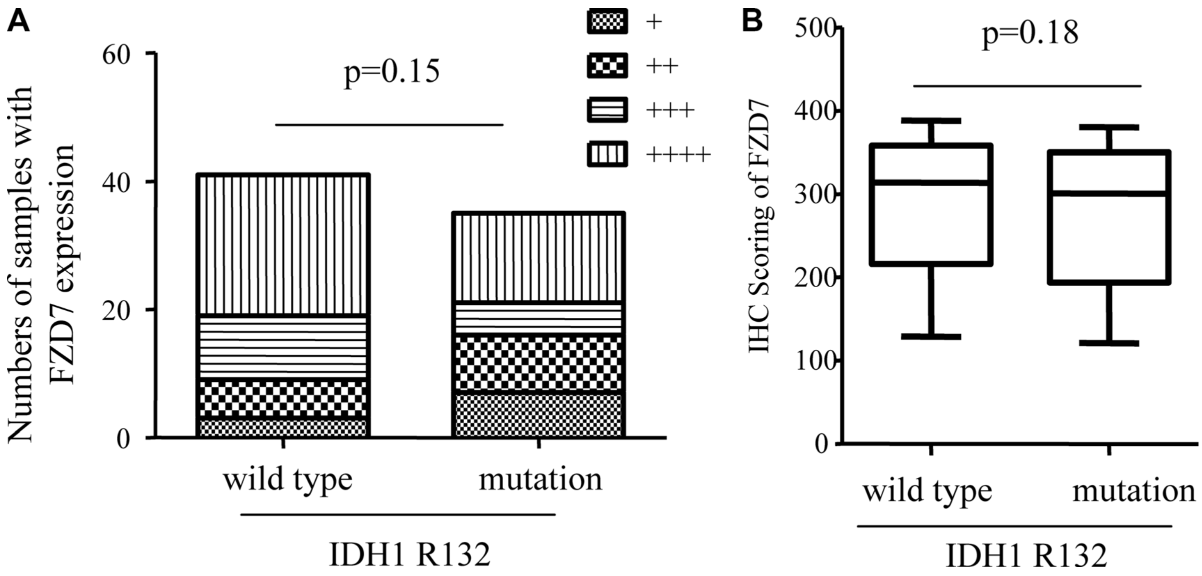

Supplementary Figure S1: The expression of FZD7 in patients with IDH wild type or mutation. **(A)** Expression levels of FZD7 were scored semi-quantitatively based on the percentage of positive cells according to the following scale: +, < 25%; ++, 25–49%; +++, 50–74%; and ++++, 75–100%. Scale bars = 50 µm. *p* values were calculated with a two-sided Fisher's exact test. **(B)** Expression levels of FZD7 were scored by H-score.

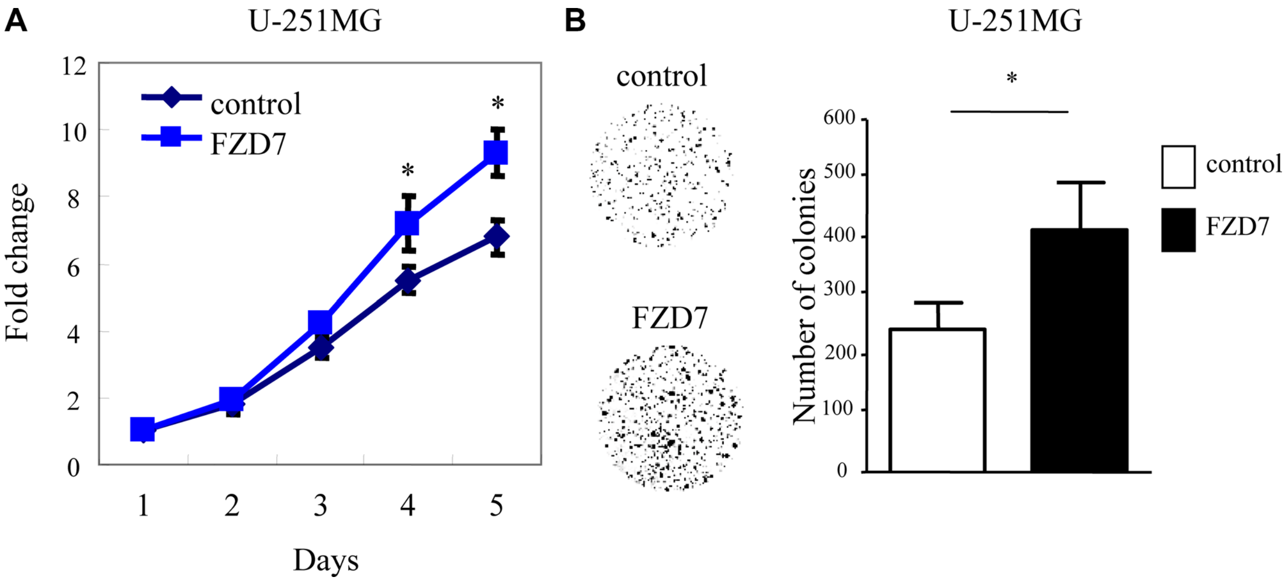

Supplementary Figure S2: FZD7 promotes cell proliferation in U-251MG cells. **(A, B)** MTT growth assays (A) and colony formation assay (B) of U-251MG cells transfected either with FZD7 or control plasmids. *$p < 0.05$.

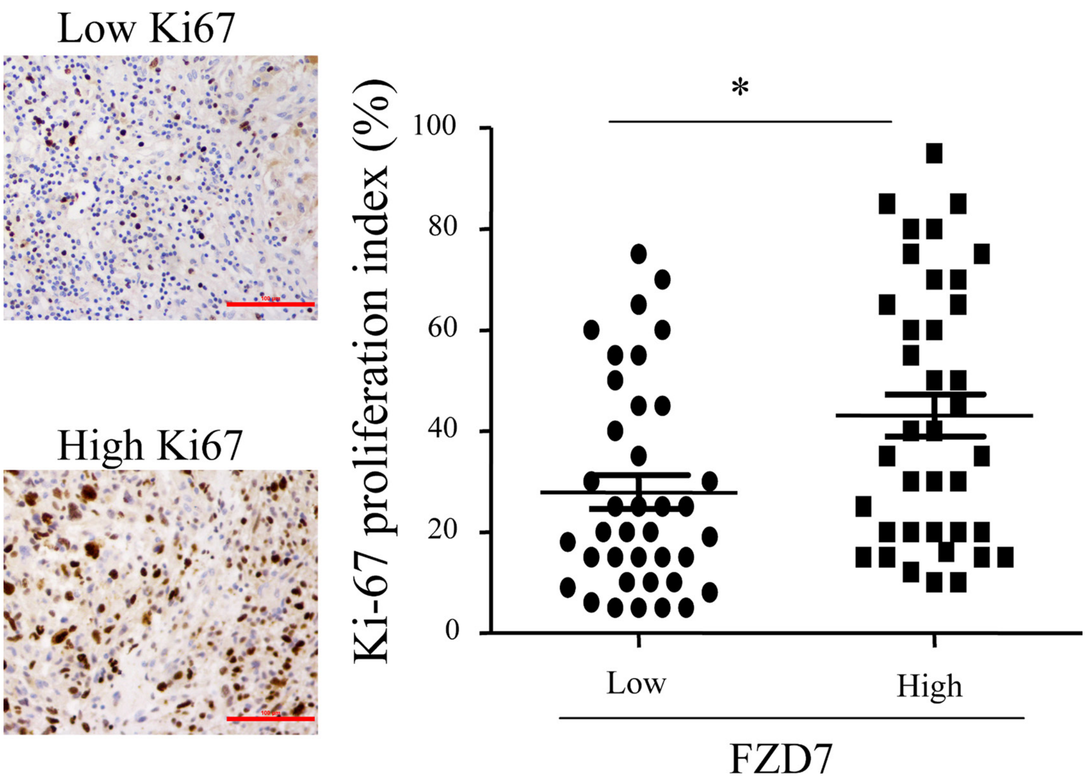

Supplementary Figure S3: Ki-67 proliferation index (%) was evaluated in glioma patients with FZD7 high or low expression. Scale bars = 100 μm. *, $p < 0.05$.

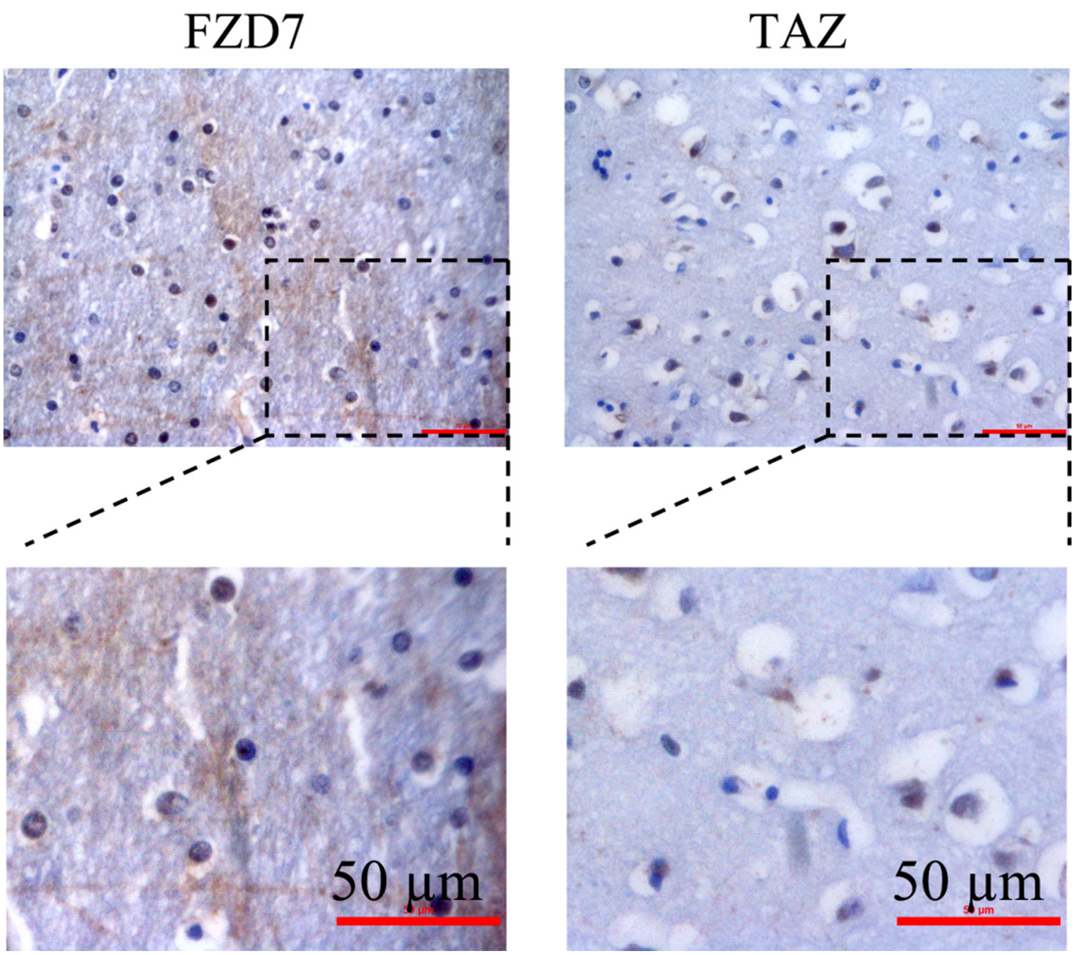

Supplementary Figure S4: The representative example of FZD7 and TAZ staining in the adjacent non-tumor brain tissue. Scale bars = 50 µm.

Supplementary Table S1: The genes that co-expressed with FZD7 in glioblastoma. See Supplementary_Table_S1.
