# Supplementary material for: Overexpression of FZD7 promotes glioma cell proliferation by upregulating TAZ
Source: Oncotarget. 2016 Nov 11;7(52):85987–99. doi: 10.18632/oncotarget.13292 (PMC5349891; doi:10.18632/oncotarget.13292)
Supplement: Supplementary file 2 [file oncotarget-07-85987-s002.doc]

**Supplementary Table 1. The genes that co-expressed with FZD7 in glioblastoma.**

| **Probeset** | **Gene symbol** | **R (﹥0.5 or ﹤-0.5)** | **p value** |
| --- | --- | --- | --- |
| 203706_s_at | FZD7 | 1 | 0 |
| **202133_at** | **TAZ** | **0.664** | **5.60E-66** |
| 203739_at | ZNF217 | 0.643 | 1.17E-60 |
| 209129_at | TRIP6 | 0.632 | 3.71E-58 |
| 200791_s_at | IQGAP1 | 0.627 | 5.08E-57 |
| 208789_at | PTRF | 0.621 | 1.09E-55 |
| 201012_at | ANXA1 | 0.614 | 4.31E-54 |
| 219973_at | ARSJ | 0.589 | 1.43E-48 |
| 207643_s_at | TNFRSF1A | 0.582 | 2.70E-47 |
| 221898_at | PDPN | 0.578 | 2.01E-46 |
| 218983_at | C1RL | 0.57 | 7.71E-45 |
| 219616_at | ACSS3 | 0.568 | 2.03E-44 |
| 201215_at | PLS3 | 0.565 | 7.16E-44 |
| 200986_at | SERPING1 | 0.564 | 7.12E-44 |
| 203729_at | EMP3 | 0.561 | 2.87E-43 |
| 212067_s_at | C1R | 0.56 | 4.24E-43 |
| 217739_s_at | NAMPT | 0.559 | 6.74E-43 |
| 202894_at | EPHB4 | 0.558 | 1.12E-42 |
| 213373_s_at | CASP8 | 0.558 | 8.95E-43 |
| 213342_at | YAP1 | 0.555 | 2.88E-42 |
| 208659_at | CLIC1 | 0.554 | 5.38E-42 |
| 208161_s_at | ABCC3 | 0.553 | 6.01E-42 |
| 203704_s_at | RREB1 | 0.551 | 1.37E-41 |
| 212063_at | CD44 | 0.55 | 2.66E-41 |
| 218802_at | CCDC109B | 0.55 | 1.93E-41 |
| 204646_at | DPYD | 0.545 | 1.42E-40 |
| 218424_s_at | STEAP3 | 0.544 | 2.56E-40 |
| 209970_x_at | CASP1 | 0.543 | 3.31E-40 |
| 202990_at | PYGL | 0.542 | 5.92E-40 |
| 217967_s_at | FAM129A | 0.54 | 1.45E-39 |
| 205559_s_at | PCSK5 | 0.535 | 1.04E-38 |
| 37408_at | MRC2 | 0.531 | 4.73E-38 |
| 221024_s_at | SLC2A10 | 0.529 | 9.32E-38 |
| 201136_at | PLP2 | 0.525 | 5.10E-37 |
| 208636_at | ACTN1 | 0.524 | 5.59E-37 |
| 201412_at | LRP10 | 0.523 | 8.13E-37 |
| 205499_at | SRPX2 | 0.523 | 8.28E-37 |
| 208949_s_at | LGALS3 | 0.522 | 1.13E-36 |
| 202729_s_at | LTBP1 | 0.519 | 3.53E-36 |
| 208816_x_at | ANXA2P2 | 0.519 | 3.58E-36 |
| 201645_at | TNC | 0.518 | 4.98E-36 |
| 205579_at | HRH1 | 0.518 | 4.97E-36 |
| 214853_s_at | SHC1 | 0.518 | 5.49E-36 |
| 221773_at | ELK3 | 0.518 | 5.44E-36 |
| 218627_at | DRAM1 | 0.517 | 7.72E-36 |
| 200600_at | MSN | 0.516 | 8.76E-36 |
| 215870_s_at | PLA2G5 | 0.515 | 1.69E-35 |
| 206580_s_at | EFEMP2 | 0.513 | 2.90E-35 |
| 201324_at | EMP1 | 0.511 | 5.59E-35 |
| 212501_at | CEBPB | 0.509 | 1.12E-34 |
| 212586_at | CAST | 0.509 | 1.45E-34 |
| 218035_s_at | RBM47 | 0.505 | 5.21E-34 |
| 201260_s_at | SYPL1 | 0.504 | 6.85E-34 |
| 209306_s_at | SWAP70 | 0.504 | 6.87E-34 |
| 221900_at | COL8A2 | 0.504 | 6.57E-34 |
| 213293_s_at | TRIM22 | 0.503 | 8.57E-34 |
| 217730_at | TMBIM1 | 0.503 | 1.11E-33 |
| 218418_s_at | KANK2 | 0.502 | 1.48E-33 |
| 209396_s_at | CHI3L1 | 0.501 | 2.06E-33 |
| 210427_x_at | ANXA2 | 0.501 | 1.77E-33 |
| 204165_at | WASF1 | -0.501 | 1.77E-33 |
| 200783_s_at | STMN1 | -0.502 | 1.23E-33 |
| 211071_s_at | MLLT11 | -0.503 | 9.65E-34 |
| 213664_at | SLC1A1 | -0.503 | 1.04E-33 |
| 213269_at | ZNF248 | -0.504 | 6.90E-34 |
| 201952_at | ALCAM | -0.505 | 4.61E-34 |
| 210721_s_at | PAK7 | -0.506 | 3.21E-34 |
| 206190_at | GPR17 | -0.508 | 1.75E-34 |
| 210341_at | MYT1 | -0.509 | 1.44E-34 |
| 209839_at | DNM3 | -0.514 | 2.14E-35 |
| 218097_s_at | CUEDC2 | -0.514 | 2.17E-35 |
| 204411_at | KIF21B | -0.519 | 4.44E-36 |
| 219732_at | LPPR1 | -0.528 | 1.19E-37 |
| 212309_at | CLASP2 | -0.531 | 4.29E-38 |
| 207723_s_at | KLRC3 | -0.546 | 1.17E-40 |
| 202967_at | GSTA4 | -0.565 | 6.96E-44 |
| 206039_at | RAB33A | -0.613 | 7.19E-54 |
